# Modeling the impact of anthrax vaccination on buffalo outbreak dynamics in northern Vietnam

**DOI:** 10.1016/j.onehlt.2025.101294

**Published:** 2025-12-10

**Authors:** Francisca Javiera Rudolph, Tan Minh Luong, Thai My Do, Van Binh Trinh, Ba Uyen Pham, Minh Dat Hoang, Anh Hung Pham, Van Truong Lu, Van Khang Pham, Thanh Long Pham, Quang Thai Pham, Thi Thu Ha Hoang, Thi Mai Hung Tran, Juan Pablo Gomez, José Miguel Ponciano, Jason K. Blackburn

**Affiliations:** a Spatial Epidemiology and Ecology Research Laboratory (SEER Lab), Department of Geography, University of Florida, Gainesville, Florida, United States; b Emerging Pathogens Institute, University of Florida, Gainesville, Florida, United States; c National Institute of Hygiene and Epidemiology, Hanoi, Viet Nam; d Dien Bien Provincial Sub-Department of Animal Health, Dien Bien Phu, Dien Bien, Viet Nam; e Ha Giang Provincial Sub-Department of Husbandry and Animal Health, Ha Giang, Ha Giang, Viet Nam; f Lao Cai Provincial Sub-Department of Animal Husbandry and Animal Health, Lao Cai, Lao Cai Province, Viet Nam; g Cao Bang Provincial Sub-Department of Plantation and Animal Husbandry, Cao Bang, Cao Bang, Viet Nam; h Lai Chau Provincial Sub-Department of Husbandry and Animal Health, Lai Chau, Lai Chau, Viet Nam; i Son La Provincial Sub-Department of Husbandry, Animal Health and Fishery, Son La City, Son La, Viet Nam; j Department of Animal Health, Ministry of Agriculture and Rural Development, Hanoi, Viet Nam; k School of Preventive Medicine and Public Health, Hanoi Medical University, Hanoi, Viet Nam; l Department of Chemistry and Biology, Universidad del Norte, Barranquilla, Colombia; m Department of Biology, University of Florida, Gainesville, Florida, United States

**Keywords:** Simulation models, Modeling, Anthrax, Vaccination, One health, Vietnam

## Abstract

A widespread and underreported zoonosis, anthrax is a severe infectious disease of significant public health concern for humans, livestock, and wildlife. In this study, we used historical data from 1991 to 2020 from northern Vietnam and a simulation model to investigate the effects of different vaccination strategies on livestock outbreaks. We developed a novel approach combining semi-synthetic data generation and a sliding windows model fitting routine to estimate disease transmission parameters from surveillance data and address the temporal mismatch between pathogen transmission dynamics and disease reporting. Results showed that vaccination leads to a significant reduction in buffalo mortality, with reactive and increasing vaccination campaigns having the largest impact in reducing outbreak size. Reactive and decreasing vaccination campaigns initially controlled outbreaks, but mortality increased as soon as vaccination ceased, highlighting the need for sustained, long-term vaccination. In scenarios where populations had high natural immunity, the impact of vaccination was less pronounced, though still evident, suggesting that prioritizing vaccination efforts for more susceptible populations may provide a greater return on investment in outbreak prevention and control. Simulation models can offer valuable insights into vaccination and control strategies, providing tools to compare and evaluate potential outbreak scenarios. Our findings underscore the value of mathematical and simulation approaches to overcome data challenges and underreporting in global disease management for anthrax and other neglected diseases. We highlight the importance of continued investment in surveillance and modeling efforts, while providing a practical approach to optimize the use of existing data in Vietnam and similar settings.

## 1 Introduction

Anthrax is a near globally distributed zoonosis caused by the gram-positive bacterium *Bacillus anthracis*, a pathogen that is transmitted indirectly through spores that survive in the environment for extended periods of time [[1], [2], [3]]. While effective vaccines are available for controlling anthrax outbreaks in livestock, the disease remains a significant public health burden affecting both humans and animals, particularly in poor rural areas [[4], [5], [6]]. Human cases tend to be associated with handling infected animals during slaughter or from consuming contaminated meat [[7], [8], [9], [10]], which typically leads to cutaneous or gastrointestinal anthrax. Cutaneous anthrax accounts for the majority of cases in humans, which, untreated can result in meningitis or death [11]. Outbreaks in livestock and wildlife can vary significantly in size involving hundreds or even thousands of animals, with massive economic losses and public health impacts [5].

In Vietnam, anthrax has been classified as a priority zoonosis for control, however, despite surveillance and vaccination efforts attempting to reduce disease burden in livestock, the number of human cases per year has remained relatively constant over the last three decades [12]. Most anthrax cases reported in the country are associated with the northern midlands and mountainous regions, with the highest incidence found in six northern provinces: Son La [13], Dien Bien [9], Lai Chau [14], Lao Cai [15], Ha Giang [16], and Cao Bang [17]. Anthrax surveillance in Vietnam currently occurs at a national level, the disease in humans was made nationally reportable in 2015 by the Circular 54/TT-BYT of Vietnam's Ministry of Health and in animals by the Circular 07/2016/TT-BNNPTNT of Vietnams' Ministry of Agriculture and Rural Developed in 2016; however prevention and control strategies have been independently implemented locally at provincial scales [18,19]. Disease control measures include vaccination, carcass burning and burying, community awareness around sick and dead livestock, and delineation of risk areas to reduce exposure. Vaccination is mandatory and determined by national guidelines, however, implementation of livestock vaccination programs and responses to outbreaks are directly established by provincial veterinary offices (Provincial Sub-Departments of Animal Health) at lower administrative units such as districts (sub-province political boundaries) or communes (sub-district boundaries). Vaccination in the area is a response to an increase in the number of human or animal cases. However, challenges including inconsistent surveillance and low-quality data fail to adequately quantify disease burden, and in the absence of disease, control measures such as vaccination are gradually stopped [15,17]. Given this independent and local response to anthrax cases in each province, there is variation regarding surveillance efforts, length of the vaccination programs, and overall vaccine coverage.

Mathematical modeling approaches have played an important role in understanding anthrax dynamics with early foundational work [20,21] establishing the importance of transmission from the environment as well as from contaminated individuals. Compartmental models have been used widely with increasing complexity to incorporate effects of animal migration, population dynamics, environmental drivers, and vaccination [[22], [23], [24], [25]]. Two important limitations characterize most of the existing anthrax models. The first is the assumption of exclusively fatal infections [22,23] despite some field studies showing that grazers recover and develop some level of immunity [[26], [27], [28]]. While some recent work has incorporated recovery dynamics [29] the second limitation is maintained across these models: the use of a fixed transmission rate parameter that fails to capture the environmental seasonality driving anthrax outbreak dynamics [[30], [31], [32], [33]].

Our study builds upon the mechanistic framework developed by Gomez et al. [34] which introduced the use of a stochastic force of infection driven by environmental covariates, allowing for seasonal forcing of transmission decoupled from population dynamics. In our study, we extend this modeling framework to incorporate the effects of vaccination and natural immunity on survival rates of livestock. Additionally, we adapt the parameter estimation approach to manage the data limitations from our study area using historical anthrax surveillance data from northern provinces in Vietnam. Additionally, we propose a semi-synthetic data generation approach and a sliding windows routine to overcome challenges in the temporal mismatch between surveillance data time scales and pathogen transmission dynamics when performing parameter estimation. We exemplify how mathematical modeling can play a key role in overcoming some of the data challenges associated with neglected diseases and yet provide useful insights on the effects of control strategies, such as vaccination, under simulated scenarios.

## 2 Methods

### 2.1 Human and animal anthrax data

**Fig. 1:**
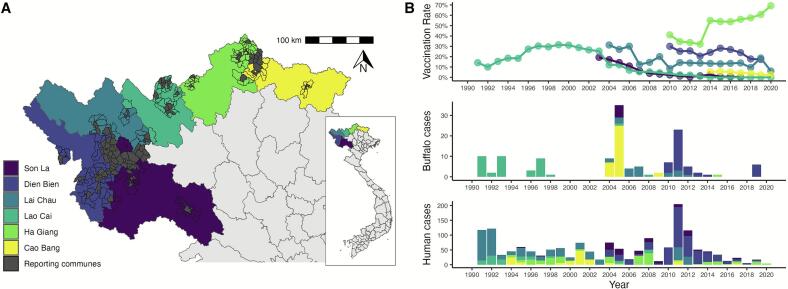
Overview of aggregated surveillance and vaccination data for six provinces in northern Vietnam. Surveillance data is aggregated from reporting communes (highlighted in grey), with high-risk neighboring commune boundaries outlined (A). Available data for the six provinces follows the color coding from the map and (B) includes vaccination rates (top panel), buffalo deaths confirmed with anthrax (middle panel) and human anthrax cases (bottom panel).

Surveillance data were previously collected and livestock population sizes were estimated for each of the six northern provinces, further details on calculation of vaccination rates and data can be found in previous publications [9,[13], [14], [15], [16], [17]]. We compiled an aggregated dataset to include the number of human anthrax cases, confirmed buffalo deaths caused by anthrax, and livestock vaccination rates, for each province at a yearly time scale (Fig. 1). We focused specifically on buffalo cases only as they represent one of the largest livestock populations in the northern region, and hold important cultural and economic significance [35]. Although some provinces recorded anthrax cases in other livestock species, this information was inconsistent across provinces and years; human surveillance was as more consistent across the time period as reported across previous studies, and human cases are often linked to animals cases. Additionally, livestock vaccination rates were previously calculated as functions of total number of doses administered to combined buffalo and cattle populations, and we were unable to separate buffalo-specific rates; in this paper we assume vaccination rates apply to our simulated buffalo populations. Widely, animal surveillance systems are subject to underreporting, as livestock deaths in remote areas may not be officially reported or may be misattributed to other causes. Given the strong epidemiological link between human and animal anthrax in this region, we made the conservative assumption that each reported human case was indicative of at least one animal death in the reporting region and time frame. While this represents a substantial assumption, it allows us to work with the available surveillance data, acknowledging the likely underestimation of true animal burden. When provinces reported zero buffalo cases for a given year, this represents an official report of zero cases by the surveillance agency rather than missing data, however we acknowledge that this likely reflects underreporting rather than true absence of disease.

To address the temporal mismatch between annual livestock surveillance data and the weekly transmission dynamics of our model, we leveraged the fact that most human case reports included month-of-occurrence information. We used the seasonal distribution of human cases to estimate the monthly patterns of anthrax occurrence, which we subsequently used in the semi-synthetic data generation process to simulate weekly incidence patterns for a simulated buffalo population.

### 2.2 Semi-synthetic data generation

Our study developed an extension to a previously developed compartmental model that describes transmission dynamics for an environmentally-transmitted pathogen, the SMILE model [34]. This compartmental model was originally formulated for an anthrax outbreak in farmed bison, *Bison bison bison,* ranging across a large landscape in the state of Montana (U.S.) for one year, using weekly time steps to adequately capture transmission dynamics. The general model structure and set of equations can be found in the Supplement and further details are in the original study [34].

One of the key methodological challenges we faced was the temporal mismatch between the annual surveillance data and the weekly transmission dynamics required by the SMILE model. We addressed this through a semi-synthetic data generation approach that preserved the yearly totals while distributing them across weeks within each year.

**Fig. 2:**
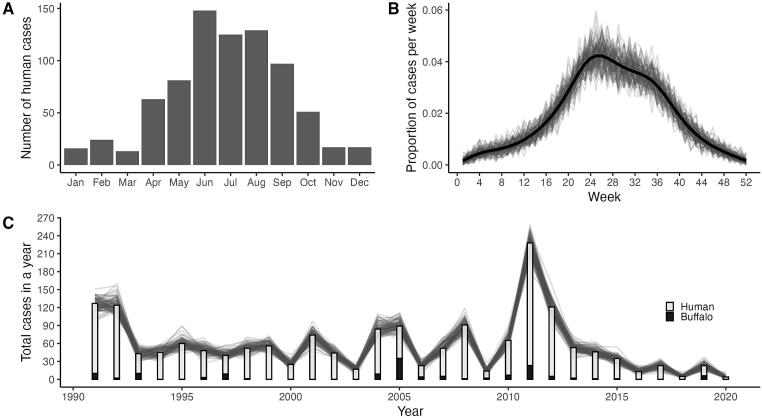
Semi-synthetic approach for data simulation that combines yearly case totals with seasonal probabilities. A) Monthly distribution of human anthrax cases combined across all provinces. B) Estimated probability of cases for each week from KDE used to rescale monthly data to a weekly time scale shown in the black line. Resulting proportion of cases per week from each simulated time series shown in the grey lines. C) Total number of aggregated buffalo and human cases per year across all provinces. Yearly totals from simulated time series represented by the grey curves.

For each year, we calculated the total number of anthrax cases from the data and then distributed these annual totals across the 52 weeks in each year using a binomial sampling process. Formally, let random variable $X_{i,s}$ represent the number of anthrax cases in week i for year s, then $X_{i,s}∼B\left(n_{s},p_{i}\right)$, where $n_{s}$ is the total number of cases in year s and $p_{i}$ is the probability of a case occurring on week i. The weekly probabilities were derived from the monthly distribution of human cases using kernel density estimation (KDE) to create a smooth probability density function for all weeks ($p_{i},i=1,2,\ldots ,52$), encompassing a full year, and ensuring that the seasonal pattern observed in human cases informed the temporal distribution of our semi-synthetic data (Fig. 2). This process generated 100 semi-synthetic weekly time series of buffalo anthrax deaths spanning 1991 to 2020, each preserving the annual case totals from surveillance data while incorporating realistic seasonal variation. These livestock time series served as input data for model fitting and parameter estimation procedures described in the following section.

### 2.3 Model parameters and estimation

Our goal in this study was to estimate key disease transmission parameters of the SMILE model using surveillance data from Vietnam, allowing us to simulate scenarios relevant to this specific context and region (see Table 1 for a summary of all the parameters in the model). The SMILE model is a compartmental model that explicitly incorporates seasonal forcing in the transmission parameter (λ). Simply stated, transmission is not a constant, but a function dependent on disease dispersion and seasonality. The full model is described in the Supplement. We focused on estimating two sets of parameters: we estimated disease dispersion parameters (τ,θ), and seasonality parameters ($b_{0},b_{1}$). Disease dispersion in the SMILE model describes how infected animals move through the landscape and create new infectious hotspots when they die, forming a “local infectious zone” (LIZ). The seasonality parameters capture how environmental conditions influence transmission rates throughout the year. To estimate these parameters, we used maximum likelihood estimation, a statistical method that finds parameter values that make the observed data most probable under the suggested model. Following the approach described in Gomez et.al [34], we treated the weekly case counts from the semi-synthetic data as observations from the L compartment in SMILE that follow a Poisson distribution. The relationship between the weekly case count data and the model predictions were formulated as $l_{t}∼\mathrm{Poisson}\left(L_{t}\right)$, where $l_{t}$ and $L_{t}$ correspond to the observed and expected number of buffalo deaths at time t, respectively. We wrote the likelihood as follows:

$$\ln\left(L\left(l;b_{0},b_{1},\tau ,\theta \right)\right)=\sum_{t=1}^{T}\ln\left(\frac{L_{t}^{l_{t}}e^{-L_{t}}}{l_{t}!}\right),$$

estimating parameters as the values that maximized that likelihood. The model was written using the statistical programming language R and we estimated the parameter values by minimizing the negative log likelihood using the optim function [36].

**Table 1:** Parameters considered in the SMILE model and their description.

Parameter	Description	Value	Source/Justification
$\lambda \left(t\right)$	Force of Infection	$P\left(X\left(a\right)\geq 1\right)=\frac{{\left(\mathrm{bE}+\theta \right)}^{\tau }-\theta^{\tau }}{{\left(\mathrm{bE}+\theta \right)}^{\tau }}$	Calculated in [34]
τ,θ	Disease dispersion effort	Estimated values in Table 2	
$b_{0},b_{1}$	Strength of seasonality	Estimated values in Table 2	
$\zeta \left(t\right)$	Probability of survival after infection	$\frac{1}{1+e^{-\left(\beta_{0}+\beta_{1}\cdot \nu_{t}\right)}}$	Extension to [34] developed in this study
Π	Periodicity of outbreaks	3 years	Periodicity in northern Viernam [12]
α	Loss of immunity	0.02	Weekly rate approximation assuming a loss of immunity after one year ∼1/52
ψ	Number of spores per carcass	1	Assumption in [34]
γ	Spore persistence rate	0.9868	Conservative estimate from [50]
$\rho \left(N_{t}\right)$	Reproduction probability	$\frac{\rho }{1+{\left(\frac{N_{t}}{K}\right)}^{10}}$	Formulated in [34]
$N_{t}$	Population size at time t	$S_{t}+M_{t}$	
ρ	Average reproduction rate	0.36	Estimated value for the region [51]
K	Carrying capacity	60,000	average buffalo population across all the reporting communes in the study area during the study period

#### 2.3.1 Sliding windows approach

**Fig. 3:**
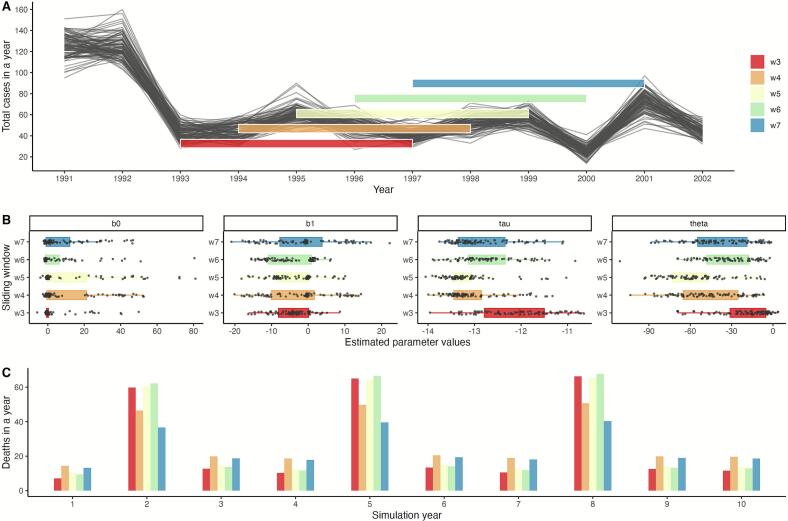
Visual example of the sliding windows model fitting routine and distribution of estimated parameters. For visualization purposes, we illustrate a subset of the semi-synthetic data and estimated parameters. A) Example of the sliding windows model fitting routine, showing five overlapping windows that capture the subset of data considered for parameter estimation (black lines). B) Boxplots of the estimated parameters for each of the time series within the window color matched to A). Every point is the estimated value for each parameter in one semi-synthetic time series. The first two panels correspond to seasonality parameters, b0 and b1, and the disease dispersion parameters correspond to tau (τ) and theta (θ) in log scale. C) Total deaths per year corresponding to simulations using the mean parameter values for each of the windows represented here.

To address the variability in surveillance quality and disease patterns over time, we implemented a sliding windows parameter estimation routine. Rather than assuming fixed values for disease transmission (dispersion and seasonality parameters) across the entire 30-year study period, we divided each of the semi-synthetic time series into overlapping 5-year segments (captured weekly; 260 weeks) and estimated the set of four transmission parameters ($b_{0},b_{1},\tau ,\theta$) for each window (Fig. 3). This approach is conceptually similar to epidemiological nowcasts, where disease forecasts are continuously updated as new data becomes available [37,38]. However, in our study we applied it retrospectively to capture time-dependent variation in the parameters. This sliding windows method serves two main purposes: the first is to account for potential changes in surveillance effort or disease transmission over time, and the second is that it generates multiple parameter estimates that allow us to quantify uncertainty in our estimation, like bootstrap resampling methods but adapted to our specific data structure.

### 2.4 Vaccination and immunity scenario simulations

Using the disease transmission parameters estimated through our sliding window approach, we designed a series of simulation experiments to evaluate four different vaccination campaigns with a 10-year duration: a baseline of no vaccination (vax0), a continuous but low vaccination rate (vax1), a reactive and decreasing (vax2; reacting to a previous outbreak rather than preemptive control), and a reactive and increasing (vax3). These vaccination strategies were informed by the reported data from each of the provinces.

The simulations used the SMILE model parameterized with our estimates of disease dispersion (τ,θ) and seasonality ($b_{0},b_{1}$) parameters, ensuring that the transmission dynamics reflected the epidemiological patterns observed in Northern Vietnam. The simulated buffalo population size considered for the simulations was 60,000, which represented the average buffalo population across all the reporting communes in the study area during the study period. Simulated data for each individual province with their specific vaccination data are provided in the supplement (Fig. S1).

For each of the vaccination strategies, simulations also incorporated the effects of natural immunity in the susceptible population to represent variation in antibody titers to *B. anthracis* reported across different species [26,27]. Here, natural immunity was defined as the proportion of susceptible individuals infected with *B. anthracis* that survive after exposure to the pathogen; we focused on three levels: low, medium, and high. To implement these vaccination and immunity effects into the simulations, we modified the transmission model in the original SMILE framework as described below.

#### 2.4.1 SMILE model extension

Here, we modified the original SMILE model to incorporate the effects of vaccination into the rates at which individuals from the infected (I) compartment become immune (M) or die (L). The original SMILE model assumed a constant post-exposure survival rate (ζ = the probability of becoming immune), which determined the transition rate from the I- infected to M- immune compartments. In our extension, we have incorporated variability in the post-exposure survival rate (as caused by vaccination) by incorporating a time-dependent variable:

$$\zeta_{t}=f\left(\nu_{t}\right)=\frac{1}{1+e^{-\left(\beta_{0}+\beta_{1}\cdot \nu_{t}\right)}}$$

where post-exposure survival rate due to vaccination was modeled as a function of initial natural immunity, $\beta_{0}\;$, and vaccine-acquired immunity, $\beta_{1}$, following a logistic curve. This equation calculates the survival rate, $\zeta_{t}$, as a function of vaccination at time t, where $\nu_{t}$ represents the population-level vaccination rate.

In our simulation experiments, we focused on three levels of natural immunity corresponding to the $\beta_{0}$ parameter when vaccination level is set to zero (low = 0.3, medium = 0.5, and high = 0.88), and derived $\beta_{1}$ algebraically by assuming a maximum survival probability of 0.97 under 100 % vaccination based on the based on the available literature [39]. Each of the vaccination strategies described above was used for the vaccination rate, $v_{t}$, and given that vaccinations were only administered once per year, vaccination rate was fixed for any year t. The resulting simulations of weekly time series for number of animal anthrax deaths (L in SMILE) were aggregated by year for each of the vaccination and immunity scenario combination.

## 3 Results

The complete surveillance dataset included human case numbers, buffalo deaths, and livestock vaccination rate for each of the six provinces from 1991 to 2020. Livestock vaccines are administered once annually mainly to buffalo and cattle. Vaccination trends are shown in Fig. 1. Lao Cai province displayed a reactive and decreasing 23-year long vaccination campaign reaching a maximum of 31.5 % vaccination. A reactive and increasing campaign was observed for Ha Giang province, starting in year 2010 and reaching a maximum of 69.2 % for the last data point in 2020. Vaccination rates were previously reported for each province and calculated as the total number of vaccine doses over the total population of buffalo and cattle in the province [9,[13], [14], [15], [16], [17]]. Variation in case numbers across provinces were observed, with human cases being higher than buffalo deaths every year. The biggest outbreak of human cases in 2011 coincided with a large number of buffalo deaths, with most reported from Dien Bien province.

The monthly distribution of human anthrax cases peaked between June and August (Fig. 2A), and the proportion of cases per week across the semi-synthetic time series closely followed this trend with peak probabilities between weeks 24 and 32 (Fig. 2B). Similarly, the yearly totals for each of the simulated time series paralleled the total number of anthrax cases (human and buffalo combined across all provinces), supporting the semi-synthetic time series generation matching the epidemiological data at different time scales (Fig. 2C).

A total of twenty-six sliding windows were applied across the one hundred semi-synthetic time series data, yielding 2600 subsets of the data for which the transmission parameters were estimated (Fig. 3). Mean parameter values were calculated for each window, and overall mean and standard errors for each parameter are reported in Table 2.

**Table 2:** Estimated transmission parameters with mean and standard error calculated from the aggregate parameter values for each of the sliding windows.

Estimated Parameter	Mean	Standard Error
$b_{0}$	14.09	2.8
$b_{1}$	−4.11	9.e-01
τ	1.42e-05	6.2e-06
θ	1.01	6.7e-01

**Fig. 4:**
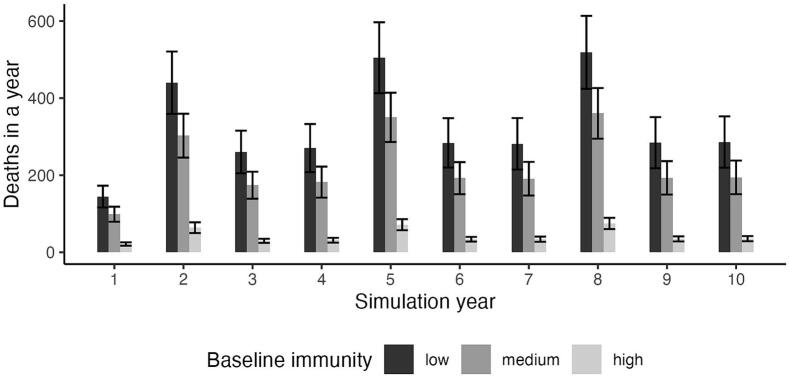
No vaccination baseline values for simulations under three different levels of natural immunity. Error bars are the result of multiple simulations under different sets of parameters from the sliding windows estimation routine, with bar height showing the mean value across all simulations. For all levels of immunity, outbreaks are observed with higher number of deaths every three years, with the largest outbreak numbers corresponding to low immunity scenarios. Variation in natural immunity levels is expected when considering different animal populations or host species.

Outbreak trends from baseline simulations with no vaccination showed increases in the number of yearly animal deaths every three years (on years 2, 5, and 8), with scenarios of low immunity consistently having the largest number of deaths (Fig. 4). Standard error bars showed minimal overlap between low and medium immunity simulations, and no overlap with high immunity simulations. During outbreak years, the mean ± SE number of animal deaths were 488 ± 51 for low immunity, 338 ± 36 for medium immunity, and 70 ± 8 for high immunity simulations.

**Fig. 5:**
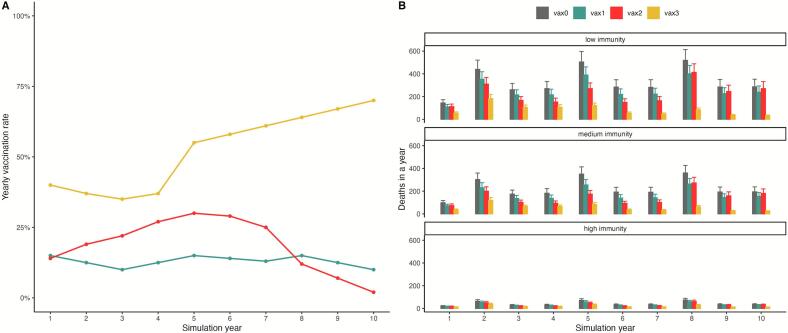
The effects of different vaccination strategies for a simulated animal (buffalo) population in the context of Vietnam data. A) Vaccination campaigns derived from strategies used by different Vietnam provinces. A reactive and increasing vaccination campaign (vax3 in yellow) follows a similar trajectory to Ha Giang province. A reactive and decreasing campaign (vax2 in red) is analogous to the vaccination pattern of Lao Cai Province. A low but continuous vaccination campaign (vax1 in teal) follows the strategy shown by the other four provinces. No vaccination is shown in grey (vax0). Specific province simulations are reported in the supplement. B) Under low immunity scenarios, differences among vaccination strategies are clear, with a campaign of increasing vaccination rates achieving the lowest mortality numbers. Outbreaks are observed every three years, although differences between outbreak and non- outbreak years are less clear in scenarios of high immunity. Even a low but continuous vaccination campaign can have an effect in reducing number of annual deaths compared to no vaccination (vax1); differences are less clear as natural immunity levels increase. (For interpretation of the references to color in this figure legend, the reader is referred to the web version of this article.)

Baseline simulations with no vaccination showed the largest numbers of animal deaths under all immunity scenarios (Fig. 5). A low but continuous vaccination strategy (vax1) displayed a lower number of yearly deaths than the no vaccination baseline, but followed the same temporal pattern with increasing number of deaths in years 2, 5, and 8. The number of deaths in the reactive and declining vaccination strategy (vax2) rapidly reached non-vaccinated scenario levels as vaccination rates approached zero in years 9 and 10, suggesting no long-term vaccination coverage and the need for a continued vaccination campaign to prevent anthrax outbreaks in simulated animal populations. In contrast, the reactive and increasing vaccination strategy (vax3), despite showing the same temporal pattern of larger outbreaks every three years, achieves the lowest number of deaths in the highest vaccination year with 31 ± 6 for low immunity, 23 ± 5 for medium immunity, and 11 ± 2 for high immunity simulations (Fig. 5B).

**Fig. 6:**
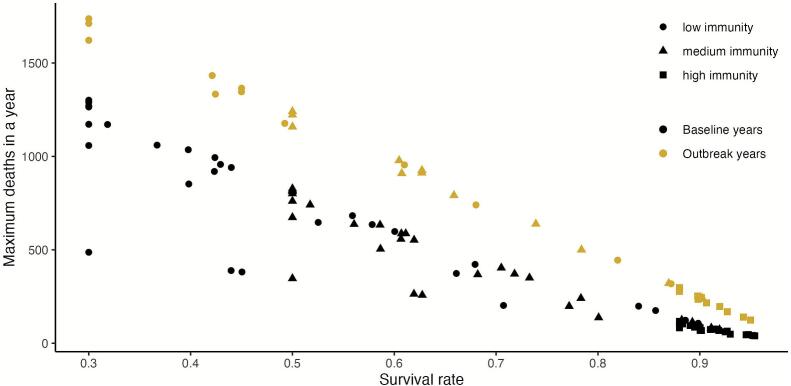
Relationship between survival rate of the population and the maximum number of animal deaths during outbreak and baseline years across all simulations. Individual data points correspond to the maximum number of deaths in a simulation for all combinations of vaccination strategies and natural immunity. Increases in survival lead to a decrease in number of deaths per year. As survival levels increase, whether by higher starting immunity or as a product of increased vaccination, the difference between number of deaths in outbreak and non-outbreak years is reduced.

Increasing the probability of survival for host populations meant a reduction in the numbers of anthrax deaths per year, whether this increase in survival was due to the population's natural immunity or in combination with vaccination (Fig. 6). There was a clear separation in the maximum number of deaths per year between baseline years and outbreak years, with this difference being reduced as survival rate was increased; the overall range of maximum deaths for outbreak years was from 124 to 1734 and for baseline years it ranged between 40 and 1290, with the lowest values of the range corresponding to simulations with higher survival probabilities.

## 4 Discussion

Building on, and expanding, an existing mathematical model for anthrax transmission, we conducted a comprehensive simulation study on the role of vaccination in reducing buffalo anthrax outbreaks using a 30-year dataset from six provinces in Northern Vietnam. We also examined variation in natural host immunity using this same approach and in combination with vaccination efforts. Our models showed that vaccination resulted in substantial reductions in the magnitude of anthrax outbreaks across all tested scenarios of immunity and vaccination levels, with even low vaccination rates (vax 1 < 25 %) achieving meaning reductions in animal deaths, especially with low and medium immunity scenarios. This finding supports the effectiveness of anthrax vaccination to reduce disease burden [6,40]. The effectiveness of low but consistent vaccination strategies has important implications for livestock disease control in resource-limited settings as most provinces in this study sustained vaccination rates below or close to 30 %. Our results suggest that consistent low-level vaccination programs may be more sustainable and effective than intensive campaigns that risk ending due to resource constraints or perceived disease elimination. Previous studies show that decreases in livestock vaccination lead to an increase in outbreaks [16,[40], [41], [42]].

Although anthrax is commonly considered a disease with extremely high mortality, serologic surveillance in wildlife has shown that a wide range of species are often exposed to the pathogen and mortality rates vary widely [26,27]. Variation in host susceptibility holds for domestic livestock species as well, with suids and bovids more resistant than ovids and equids, though more work is needed in this area [3,27]. Though it is noteworthy that lethal dose data remain limited with few recent studies [43]. Our simulations considered varying levels of immunity, given that in Vietnam multiple livestock species are reported during outbreaks, but their susceptibility varies [15]; there is also variation in vaccinating non-bovids across provinces. Our simulation results suggest that prioritizing continuous vaccination over reactive and declining strategies might be the more effective at reducing disease related animal death. Additionally, we observed that as levels of immunity increased, the effects of vaccination were not as pronounced, and the difference between outbreak years and baseline years was not as large.

The spatial distribution of *B. anthracis* is limited on local landscapes by a combination of environmental and climatic conditions [3,44]. The SMILE model and simulations incorporated a seasonal component to anthrax transmission, with parameter values estimated from Vietnam's surveillance data that capture the temporal patterns of anthrax transmission observed in this region. While our seasonal parameters effectively represent disease seasonality, they do not yet explicitly link to specific environmental drivers such as soil composition or vegetation characteristics that are known to influence spore survival [30,31]. Our knowledge of *B. anthracis* highlights the importance of these environmental factors, which when linked to climatic and seasonal patterns are associated with disease transmission and outbreaks [33]. Future efforts will focus on expanding the transmission model in SMILE to incorporate explicit environmental and soil characteristics. This environmental integration is important because, coupled with outbreak information, it could help inform vaccination strategies and prioritize areas where host populations overlap with areas of environmental suitability for anthrax [15,45,46].

This study provides a novel approach to overcome challenges associated with parameterizing complex models with insufficient data. As with many neglected zoonoses, the data available for Vietnam likely represents and underestimation of the true burden of disease [9,13]. Underreporting due to limited resources for surveillance and testing is common, however, we also need to consider that livestock is a valuable asset for people in Vietnam, and reporting deaths due to disease might carry negative consequences for them, such as the inability to sell the meat [9]. Given that risk factors for human anthrax tend to be associated with slaughtering diseased animals or eating and handling contaminated meat [7], we assumed that all human cases corresponded to an animal death in a 1:1 proportion, an assumption that should be investigated in future modeling efforts. In a recent study in Son La province, phylogenetic analysis of human and animal strains indicated undetected animal cases that were discovered with additional epidemiological investigation confirming the need for revisiting this assumption [47]. However, this assumption allowed us to get better predictions for the number of animal deaths in the sample population based on reporting communes. The semi-synthetic data generation and model fitting approach described in this study also provides a valuable solution to address the common temporal mismatch between yearly reporting of zoonotic diseases and the days to week transmission dynamics of the pathogens.

We effectively used a combination of simulation and statistical inference withhistorical surveillance data to predict livestock anthrax burden under various vaccination scenarios and natural immunity levels. Similar to our results, other modeling approaches have also employed numerical simulations that show increasing vaccination rates reduce disease and have the potential to eradicate the disease from the ecosystem [24,48]. Some modeling approaches have also found that a combination of control strategies such as appropriate carcass disposal and vaccination can greatly reduce disease spread [49]. Nonetheless, several limitations in our study should be acknowledged. Our 1:1 assumption between human cases and animal deaths requires validation through more comprehensive surveillance studies. The seasonality parameters in our models capture time-dependent patterns in the surveillance data, but do not explicitly represent soil and climatic drivers known to influence anthrax ecology. Finally, while our focus on buffalo is justified by their importance in Northern Vietnam's agricultural landscape, it may not fully represent transmission dynamics across all livestock species in the region.

A persistent challenge in epidemiological modeling is effectively translating data into knowledge that aids policy making or control strategies. In this study we presented a methodology to maximize the use of available surveillance data, despite underreporting and high uncertainty in true case numbers. Addressing underreporting through improved surveillance systems coupled with dynamic modeling approaches have the potential to accurately inform and effectively design control and vaccination campaigns that will minimize animal deaths and subsequently reduce disease risk to humans.

## 5 Conclusions

This study used novel modeling techniques to simulate anthrax outbreak scenarios under different vaccination control strategies using a historical 30-year surveillance dataset from Vietnam. We found that continuous livestock vaccination is the most beneficial strategy in minimizing anthrax outbreaks, even when vaccination levels are low.

## Data sharing

Specific outbreak data cannot be shared publicly due to institutional review boards (IRB) restrictions in Vietnam and the USA. ‘De-identified’ data are available from the National Institute of Hygiene and Epidemiology (NIHE) institutional data access/Ethics Committee for researchers who meet the criteria for access to confidential data. Limited spatial data may be available upon reasonable request to NIHE (nihe@nihe.org.vn) pending IRB review and approval. Simulation data are available from NIHE upon request.

## CRediT authorship contribution statement

**Francisca Javiera Rudolph:** Writing – review & editing, Writing – original draft, Visualization, Validation, Software, Methodology, Formal analysis, Conceptualization. **Tan Minh Luong:** Writing – review & editing, Writing – original draft, Visualization, Validation, Methodology, Investigation, Formal analysis, Data curation, Conceptualization. **Thai My Do:** Resources, Investigation, Data curation. **Van Binh Trinh:** Resources, Investigation, Data curation. **Ba Uyen Pham:** Resources, Investigation, Data curation. **Minh Dat Hoang:** Resources, Investigation, Data curation. **Anh Hung Pham:** Resources, Investigation, Data curation. **Van Truong Lu:** Resources, Investigation, Data curation. **Van Khang Pham:** Writing – review & editing, Resources, Project administration, Investigation, Data curation. **Thanh Long Pham:** Writing – review & editing, Resources, Project administration, Investigation, Data curation. **Quang Thai Pham:** Writing – review & editing, Supervision, Resources, Project administration, Methodology, Investigation, Data curation. **Thi Thu Ha Hoang:** Writing – review & editing, Supervision, Resources, Project administration, Investigation. **Thi Mai Hung Tran:** Writing – review & editing, Supervision, Resources, Project administration, Investigation. **Juan Pablo Gomez:** Software, Methodology, Conceptualization. **José Miguel Ponciano:** Writing – review & editing, Supervision, Software, Methodology, Formal analysis, Conceptualization. **Jason K. Blackburn:** Writing – review & editing, Writing – original draft, Visualization, Supervision, Resources, Project administration, Methodology, Investigation, Funding acquisition, Formal analysis, Conceptualization.

## Declaration of competing interest

The authors declare that they have no known competing financial interests or personal relationships that could have appeared to influence the work reported in this paper.

## Data Availability

Data will be made available on request.

## Appendix A Supplementary data

Supplementary material: SMILE Model primer and province-specific simulations. Image 1
