# Supplementary material for: Modeling the impact of anthrax vaccination on buffalo outbreak dynamics in northern Vietnam
Source: One Health. 2025 Dec 10;22:101294. doi: 10.1016/j.onehlt.2025.101294 (PMC12769850; doi:10.1016/j.onehlt.2025.101294)
Supplement: Supplementary file 1 — Supplementary material: SMILE Model primer and province-specific simulations. [file mmc1.docx]

**SUPPLEMENT**

SMILE model: A primer

The SMILE model is a flexible compartmental model developed to describe weekly anthrax dynamics, or other pathogen systems where the pathogen is indirectly transmitted through the environment (Gomez *et al.* 2018). The model consists of a set of difference equations where subsets of the population move between compartments (S – susceptible, M- Immune, I – Infected, L – Dead or Local Infectious Zone which generate spores in the E - Environment) at rates that depend on the host population or parameters specific to the system (set of equations 1 in Gomez et.al. (Gomez *et al.* 2018)). The original set of recursions is below:

$$S_{t}= \sigma\left( 1-\lambda\right)S_{t-1}+ \sigma\left( \alpha M_{t-1} \right),$$

$$M_{t}=\zeta I_{t-1}+\sigma\left( \left( 1-\alpha\right)M_{t-1} \right),$$

$$I_{t}=\lambda S_{t-1},$$

$$L_{t}=\left( 1-\zeta\right)I_{t-1},$$

$$E_{t}=\psi L_{t-1}+\gamma E_{t-1}.$$

In the deterministic form, this model shows the expected number of individuals in each of the compartments at a given time $t$ based on the number of individuals at the previous time point in $t-1$; in this case the time scale is one week. Using this set of recursions, we simulate disease dynamics by providing parameters for infection probability ($\lambda$), non-disease mortality ($\sigma$), probability of becoming immune after exposure ($\zeta$), number of spores introduced into the environment by a carcass ($\psi$), and spore death rate or loss of virulence ($\gamma$). The model was tested and developed based on a bison *(Bison bison bison*) outbreak in Montana, USA, in 2008 (Gomez *et al.* 2018). The parameters described in the set of recursions above can be static (do not change over time) for the simplest cases. However, the infection probability, $\lambda(t)$, can also be described as a function of time, derived by modeling successful disease transmission as a pure birth process dependent on the seasonal transmission probability, $b(t)$, the number of spores in the environment ($E_{t}$), and the pathogen’s dispersion (modeled using parameters $\theta$ and $\tau$ which describe the process of an animal becoming infected, moving, and then becoming a LIZ):

$$\lambda\left( t \right)=\frac{{(bE_{t}+\theta)}^{\tau}- \theta^{\tau}}{{(bE_{t}+\theta)}^{\tau}},$$

with,

$$b\left( t \right)= e^{\left( b_{0}\left( 1+b_{1}\left( \cos\frac{2\pi t}{\Pi} \right) \right) \right)}.$$

The derivation of these equations and further details on this model can be found in the original publication of the SMILE model can be found in Gomez et.al. (2018).**Supplementary figures**

**
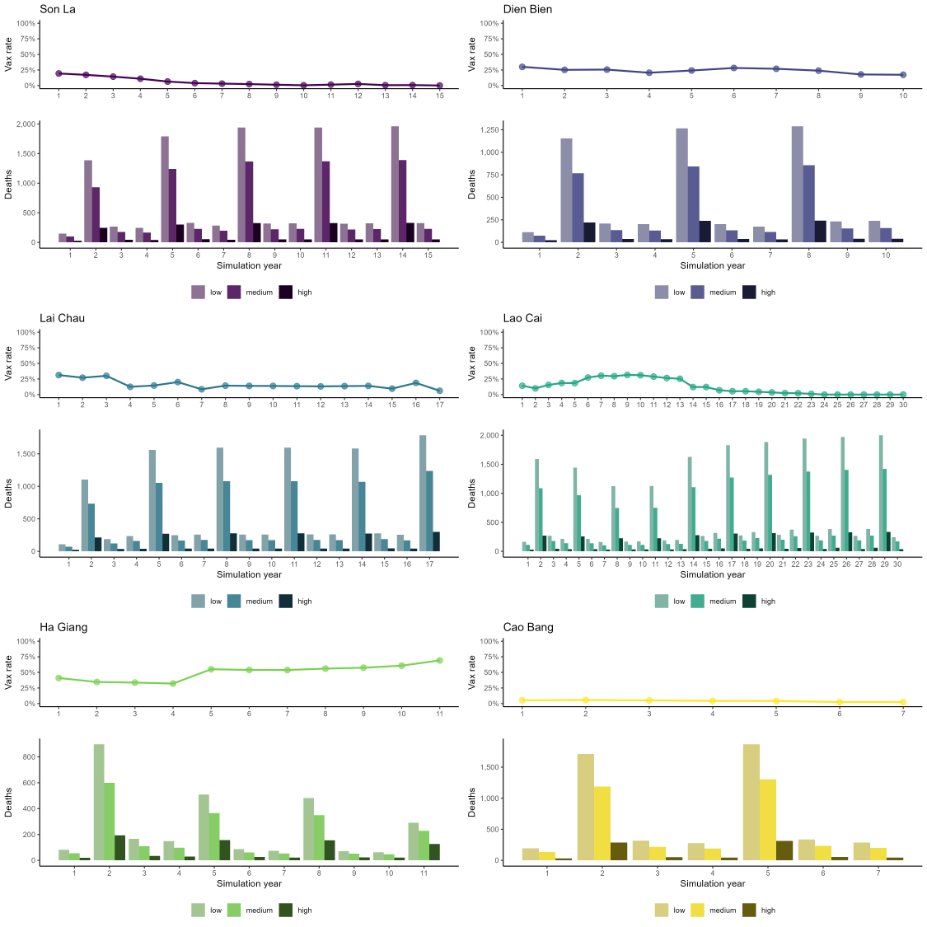
**

**Supplementary Figure 1. Province-specific simulations under their reported vaccination strategies for three different levels of natural immunity.** Increasing vaccination rates, such as Ha Giang province, shows a decrease in the number of yearly deaths across all levels of immunity. In cases where vaccination is reduced or stopped, an increase in yearly deaths is observed, such as Lao Cai province. Other provinces show vaccination levels that follow a continuous and low vaccination strategy, which contributes to a decrease in number of deaths compared to no vaccination baselines.​
